# A randomised trial of treating fibroids with either embolisation or myomectomy to measure the effect on quality of life among women wishing to avoid hysterectomy (the FEMME study): study protocol for a randomised controlled trial

**DOI:** 10.1186/1745-6215-15-468

**Published:** 2014-11-29

**Authors:** Klim McPherson, Isaac Manyonda, Mary-Ann Lumsden, Anna-Maria Belli, Jon Moss, Olivia Wu, Lee Middleton, Jane Daniels

**Affiliations:** Nuffield Department of Obstetrics and Gynaecology, University of Oxford, Women’s Centre, John Radcliffe Hospital, Oxford, OX3 9DU UK; Department of Obstetrics and Gynaecology, St George’s Hospital, Blackshaw Road, Tooting, London, SW17 0QT UK; Department of Reproductive and Maternal Medicine, Glasgow Royal Infirmary, Walton Building, 84 Castle Street, Glasgow, G4 0SF UK; Department of Interventional Radiology, Gartnavel General Hospital, 1053 Great Western Road, Glasgow, G12 0YN UK; Institute of Health and Wellbeing, University of Glasgow, 1 Lilybank Gardens, Glasgow, G12 8RZ UK; Birmingham Clinical Trials Unit, University of Birmingham, Birmingham, B15 2TT UK

**Keywords:** Uterine fibroids, UAE, Myomectomy, Quality of life, Menstrual blood loss, Ovarian reserve

## Abstract

**Background:**

Uterine fibroids are the most common tumour in women of reproductive age. By the time they are 50-years-old around 80% of women will have developed one. Of these, around half will experience symptoms which will impact negatively on their quality of life. Hysterectomy is the traditional treatment for women with symptomatic fibroids. For women who do not wish to undergo a hysterectomy, two invasive treatments are commonly available: myomectomy or uterine artery embolization (UAE).

**Design:**

FEMME is a pragmatic, randomised, open, multi-centre trial examining the quality of life menstruating women with symptomatic fibroids experience after treatment with UAE or myomectomy.

**Methods:**

After providing informed consent, 216 women with symptomatic fibroids from 43 NHS Hospital Trusts and Health Boards across the United Kingdom will undergo randomisation by a centralised computer system to treatment by either UAE or myomectomy. A minimisation algorithm will be used in order to balance the groups with respect to the following three parameters: the longest dimension of the largest fibroid, the number of fibroids present, and whether the woman currently desires pregnancy.

Using validated questionnaires the women’s quality of life will be compared between groups at six months, one year, two years and four years post-procedure, taking into account pre-procedure scores. An economic evaluation will be conducted alongside the trial to determine the cost-effectiveness of UAE compared with myomectomy.

Validated diaries will also be used to compare menstrual blood loss at the same time-points. The plasma concentration of Anti-Müllerian hormone (AMH), which will act as a proxy measurement of ovarian reserve, will be recorded before the woman has her procedure and then again at six weeks, six months, and twelve months afterwards. Re-intervention rates will be recorded.

**Discussion:**

The FEMME trial’s primary outcome is the quality of life women with symptomatic uterine fibroids experience two years after they have been treated with either UAE or myomectomy, as measured by the disease-specific Uterine Fibroid Symptom Quality-of-Life (UFS-QoL) questionnaire.

**Trial registration:**

Current Controlled Trials registration number: ISRCTN70772394, registered on 2 March 2013.

## Background

### Why is there a need to determine whether treatment with uterine artery embolisation or myomectomy is most efficacious in improving the quality of life of women with uterine fibroids?

Uterine fibroids are the most common tumour in women of reproductive age and increase in prevalence as the woman gets older. By the time they are 50-years-old, around 80% of women will have developed a fibroid. Approximately half of women with fibroids experience significant symptoms which can include heavy menstrual bleeding (HMB), pain on intercourse, abdominal pain, and a feeling of pressure, all of which can impact significantly on the woman’s quality of life [1].

The symptoms experienced by the woman with fibroids may vary depending on the position, size and number of fibroids. Intramural fibroids are the most common form of fibroid but are frequently asymptomatic. Subserosal fibroids, located on the outer surface of the uterus, can become very large and create feelings of bulkiness. Submucosal fibroids project into the uterine cavity and are associated with HMB. As they may distort the uterus and change the local morphology of the uterine tissue, some clinicians believe that the presence of fibroids may have a negative impact on fertility [2]. The Hospital Episode Statistics state that there were just under 31,000 Finished Consultant Episodes of women with fibroids of the uterus between 2012 and 2013 in the United Kingdom. The majority of these women were aged between 40 and 54-years-old [3].

Women with symptomatic fibroids often respond poorly to drug management or risk unacceptable side effects from hormonal preparations [4], so generally the use of these pharmaceuticals is limited to reducing the fibroid bulk and relieving the symptoms prior to surgical intervention. The traditional treatment for symptomatic fibroids is hysterectomy, but with many women associating their uterus with their femininity and sexuality, an increasing number of women with fibroids are questioning whether hysterectomy for a benign condition is necessary and many are seeking alternatives.

In the National Health Service (NHS) there are two womb-sparing procedures available to treat uterine fibroids; uterine artery embolisation (UAE) and myomectomy. In UAE, an arterial catheter is used to introduce an embolic agent into the blood vessel supplying the uterus. This blocks the blood flow to the fibroid causing it to infarct (die), so relieving the symptoms of the fibroid. Myomectomy is the surgical removal of the fibroid, and depending on the size and position of the fibroid, this can be undertaken by a variety of routes including laparoscopic, hysteroscopic, or by a laparotomy.

### Previous work comparing UAE with myomectomy

The use of UAE in women who may wish to conceive is controversial within some sections of the medical community. Some authors are concerned by the reduction in blood flow to the ovary that can occur if there are significant connections between the ovarian and uterine arteries, which may result in decreased ovarian function [5]. The extent of this decreased function, how long it is maintained for, and even if it occurs at all, is disputed [6, 7]. Whilst generally regarded as safe [8], the caution inherent in the guidelines drawn up when UAE was still in its infancy has led to a further reluctance in some circles to use UAE in women who may wish to try to conceive at some point. As a result of this, trials generally excluded women who are seeking fertility.

One small (n = 121) single centre, randomised, clinical trial from the Czech Republic compared myomectomy with UAE in women with intramural fibroids [9]. These authors concluded that UAE was less invasive, but as effective and safe as myomectomy for treating the symptoms of fibroids. The REST trial [10] randomised a total of 157 women in a 2:1 ratio between UAE and myomectomy before assessing the quality of life (QoL) at five years with the Short Form 36 (SF-36) general health survey. Secondary measures included complications, adverse events, and the need for further intervention. These authors reported that there were no significant differences in the reported QoL between the groups at five years. However, there was a significantly greater re-intervention rate after five years for treatment failure or complications in the UAE arm over the myomectomy arm. This negated the initial cost benefit of UAE over surgery at 12 months and made both treatments cost-neutral at five years [10].

A pilot study for the FEMME trial (the FUME study [11]) was conducted at St George’s Hospital, London, United Kingdom. In this study 160 women with symptomatic uterine fibroids were randomised to UAE or myomectomy and the change in their QoL, hospital stay, complication rates, and need for re-intervention were measured at one year post-procedure. The authors reported that by one year there was no difference in the improvement of the QoL women experienced in the myomectomy and UAE arm but, like the REST trial, there was a higher intervention rate in women who had undergone a UAE [11].

With increasing pressure on ever diminishing NHS budgets the cost-effectiveness is increasing in importance to commissioners and Trusts when deciding which treatment options should be available. The average length of stay in hospital post-hysterectomy is 4.4 days [10], and it is not unusual for women to require an additional three months to recuperate before returning to their normal lives. The average stay in hospital post-myomectomy is 3.6 days and, depending on the route used, the post-procedure recuperation time can be up to several weeks. With its minimally invasive nature, the mean length of hospital stay post-UAE is one day, with a return to normal life typically within a couple of weeks. Whilst superficially this may suggest a significant economic advantage for UAE, the higher re-intervention rates compared with hysterectomy may well negate any cost saving by five years [10].

## Methods/Design

### Study design

The FEMME trial is a randomised, open, multi-centre trial comparing UAE with myomectomy in women with symptomatic uterine fibroids.

### Primary outcome

The FEMME trial’s primary outcome is to measure the QoL women with symptomatic uterine fibroids experience two years after they have been treated with UAE or myomectomy, through the disease-specific Uterine Fibroid Symptom Quality of Life (UFS-QoL) questionnaire.

### Secondary outcomes

The FEMME trial’s secondary outcomes are:

1. EuroQoL EQ-5D score and visual analogue scale score;
2. Menstrual blood loss, assessed using the Pictorial Blood loss Assessment Chart (PBAC). This is a validated and well-used assessment of menstrual blood loss in women with uterine fibroids;
3. Pregnancy outcomes - pregnancy will be reported by the woman in the first instance. The research nurse or trial coordinator will collect further information with respect to timing of pregnancy, biochemical pregnancy (positive pregnancy test), incidence of miscarriage, outcome of pregnancy, gestational age at delivery or miscarriage, complications of pregnancy, and labour;
4. Adverse events - all adverse outcomes considered to be related to the study protocol or intervention will be collected. Since adverse outcomes may occur many months after intervention, these data will continue to be collected throughout the study;
5. Length of hospital stay;
6. Time to return to work or usual activity;
7. Further treatment for incomplete removal or recurrence of symptoms, including hysterectomies;
8. Ovarian reserve - blood samples will be taken from the women in each treatment arm for the measurement of the plasma levels of Anti-Müllerian hormone (AMH), a compound used as a proxy measurement of ovarian reserve, to determine if there is any difference at six weeks, six months, and twelve months post-procedure.

The time-points at which these follow-ups are performed are shown in Table 1.

**Table 1:** **Time-points at which outcomes are recorded**

Outcome measure	Prior to randomisation	Before discharge	6 weeks	6 months	1 year	2 years	4 years
UFS-QoL/EQ-5D	×			×	×	×	×
Pregnancy				×	×	×	×
Outcomes of pregnancy					As reported by participant		
PBAC	×			×	×	×	×
Fertility potential	×		×	×	×		
Resource usage (clinical)	×	×		×			
Technical success				×			
Serious adverse events					As reported by clinician/participant		
Further treatment				×	×	×	×

Legend:

UFS-QoL: Uterine Fibroid Symptoms – Quality of Life.

EQ-5D: EuroQol – 5D score and visual analogue scale score.

PBAC - the Pictorial Blood loss Assessment Chart.

With an increasing number of women seeking an alternative to hysterectomy, this timely trial will investigate the role of UAE and myomectomy in treating women with symptomatic uterine fibroids, and so inform women as to the most appropriate treatment for them.

### Recruitment

Patients will be recruited from multiple (more than 40) secondary and tertiary centres throughout the United Kingdom, including specialist centres, district general, and large teaching hospitals. Centres will be able to participate in the FEMME trial if a care pathway is present which allows eligible women presenting at the site to be randomised between UAE and myomectomy.

### Eligibility criteria

**Figure 1:**
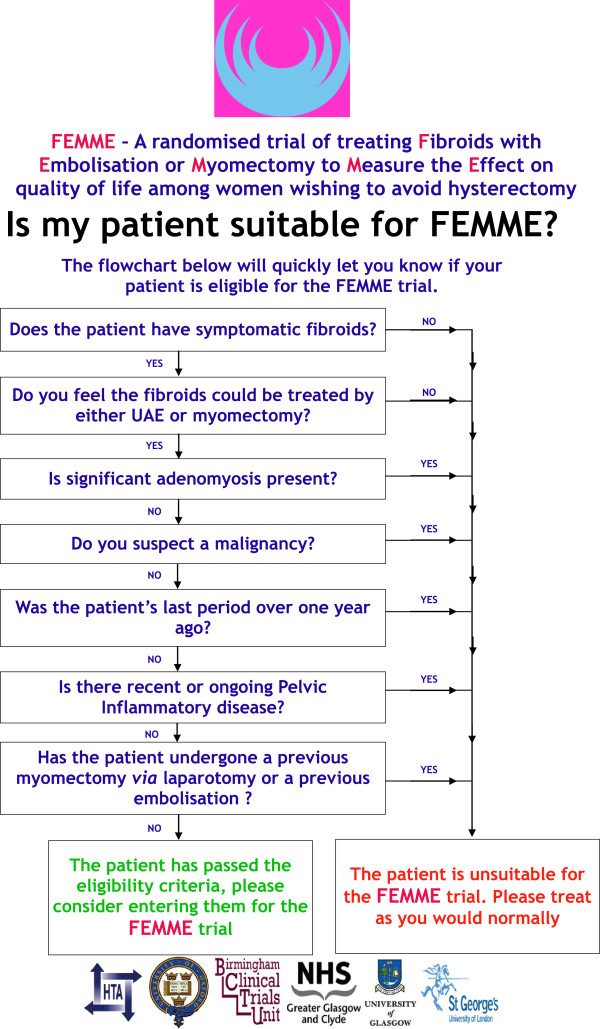
**FEMME trial eligibility flow chart.** UAE, uterine artery embolization.

A woman is eligible to be considered to take part in the FEMME trial if:

1. They have menstruated within the preceding twelve months and present with symptomatic fibroids which the reviewing clinician feels can be treated equally well with UAE or myomectomy;
2. The woman has not undergone a previous myomectomy via a laparotomy or a previous UAE;
3. They are over 18 years of age, not pregnant, and are prepared to accept a hysterectomy in an emergency;
4. There is no evidence of malignancy, pelvic inflammatory disease, or significant (in the opinion of the reviewing clinician) adenomyosis.Full eligibility criteria are shown in Figure 1.

### Patient information

Women will be identified primarily by gynaecologists in dysmenorrhea or general gynaecology clinics, but a small minority may be referred directly to an interventional radiologist. In the latter case, potential participants will need to be reviewed by a gynaecologist to ensure they are suitable for myomectomy.

Depending on local practice and staff availability, the referral letters may be screened by the research nurse who will bring to the gynaecologist’s attention those women whose symptoms are suggestive of uterine fibroids and who may be eligible to take part in the FEMME trial. After review, should the gynaecologist agree that uterine fibroids are the likely underlying cause of the described symptoms, they can request the nurse sends out a Research Ethics Committee (REC)-approved participant information sheet (PIS) and covering letter in advance. This will allow the woman to be aware of the FEMME trial before she attends the clinic for her appointment. This stage is optional and dependent on local practice and gynaecology agreement.

When the woman presents in clinic she will be reviewed by a qualified gynaecologist. Following confirmation of the presence of fibroids, if the gynaecologist feels that these will respond equally well to UAE or myomectomy, then the gynaecologist will make the initial approach to the women to make them aware of the FEMME trial. If the woman expresses an interest in taking part in the FEMME trial then an approved member of the local research team will discuss the protocol with them and answer any queries they may have.

### Consent

Consent will be obtained from any potential participant in compliance with the requirements of the REC before they are included in the trial. Any potential participant will only be approached by an approved member of the local research team. The potential participant will be given a copy of the REC-approved PIS, setting out the nature and purpose of the study, alongside the possible risks and benefits of taking part. The approved member of the local research team will fully discuss the trial with the potential participant and answer any questions they may have prior to consent being taken. Any potential participant will be informed that they can withdraw from the trial at any point without having a give a reason why. Should they wish to withdraw, then the patient will be reassured that the medical care they receive will not be affected in any way. The patient’s consent will be recorded on a REC-approved consent form and countersigned by the approved member of the local research team. Once signed and dated by all parties, the participant will be given a copy of the consent form for their own records.

### Randomisation

In order to reduce bias as far as possible, following informed consent but before randomisation, the participant will be given the validated QoL questionnaires (the UFS-QoL and the EuroQoL EQ-5D) and asked to complete them. Once completed and returned to the clinical staff, the patient’s details will be entered onto a centralised online system which will allocate the patient to their treatment (myomectomy or UAE). A minimisation algorithm will be used to ensure that the groups are balanced as evenly as possible with respect to treatment group and also the following variables: the longest dimension of the largest fibroid (under 7 cm or over 7 cm), the number of fibroids present (1 to 3, 4 to 10, over 10), and if the woman desires pregnancy (yes or no).

The number and size of the fibroids present will be determined by imaging with either: magnetic resonance imaging (MRI) (contrast enhanced and/or non-contrast enhanced), ultrasound (vaginal or trans-abdominal), or hysteroscopy.

Should a participant have been randomised on any type of imaging other than MRI then, if local capacity allows, an MRI scan will be requested prior to the patient undergoing their procedure. After randomization, blood will be drawn into two BD Vacutainer® serum separator tubes (SST, BD, The Danby Building, Edmund Halley Road, Oxford Science Park, Oxford OX4 4DQ, England) for the proxy measurement of ovarian reserve. These blood samples will be placed into a Royal Mail Safebox™ and sent to Birmingham University’s Human Tissue Repository. Upon receipt, the blood samples will be transformed into serum and stored in a secure biobanking facility at −80°C until analysis. Finally, the participant will be given a copy of the menstrual blood loss diary to take away with them and asked to complete it when they start their next period. Upon completion this blood loss diary will be returned to the trials office in a prepaid envelope provided.

### Treatment

A pregnancy test will be performed immediately prior to treatment. As FEMME is a pragmatic trial, other than the randomisation to myomectomy or UAE the treatment the woman receives is the clinician’s normal practice. This means that if the woman is randomised to a myomectomy this can be of any type including laparoscopic, hysteroscopic, or by a laparotomy. Similarly, should the woman be randomised to UAE, this is performed according to the interventional radiologist’s normal practice. Information collected at the time of the procedure will include details of the treatment received, any adverse events experienced by the participant, and the length of in-patient stay.

### Follow-up

**Figure 2:**
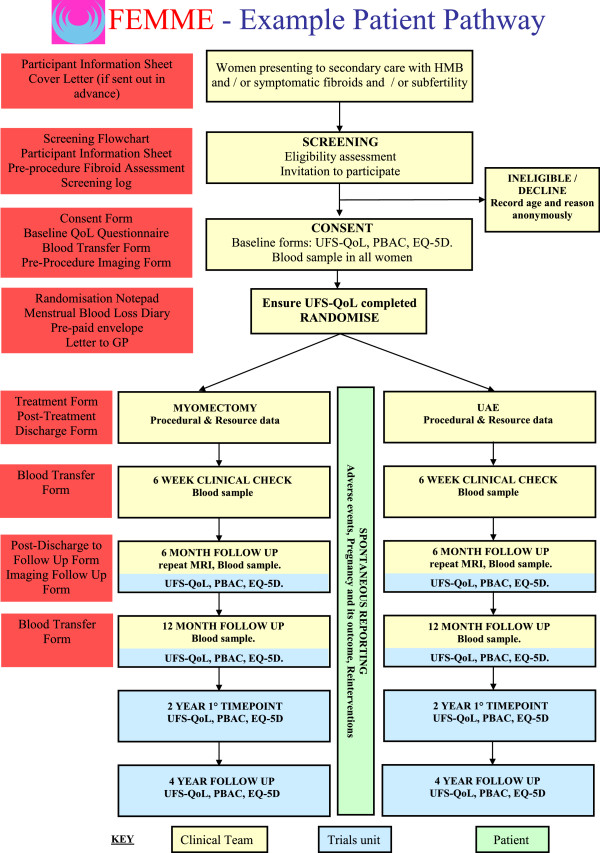
**FEMME study schema.** HMB, heavy menstrual bleeding; MRI, magnetic resonance imaging; PBAC, Pictorial Blood Loss Assessment Chart; UAE, uterine artery embolisation; UFS-QoL, Uterine Fibroid Symptom Quality-of-Life questionnaire.

The time-points when the various outcomes are recorded are shown in Table 1. At each time-point, details of any pregnancy will be collected alongside information on any resource use including:

1. The number and reason of any additional out-patient visits and/or primary care consultations;
2. Any additional procedures and inpatient stays;
3. The type and dose of any medications received;
4. Any further tests and interventions received;The information collected at each time-point is summarised in Figure 2, the study schema.

### Outcome measures

The primary outcome for the FEMME study is the health-related quality of life (HRQL) score from the UFS-QoL questionnaire recorded two years post-procedure.

#### Primary and interim analysis

HRQL scores from the UFS-QoL will be analysed using a linear model (analysis of covariance) with estimates of difference between groups adjusting for the baseline score. Results of the primary outcome score at other time-points, along with the results of other continuous-type measurements (for example, symptom severity score from the UFS-QoL questionnaire, EQ-5D and Blood Loss scores) will be analysed in a similar fashion. Further analysis of continuous measures will be undertaken using a repeated measure multilevel model to examine any differential effect over time [12].

Dichotomous measures (for example, pregnancy or re-intervention rates) will be analysed using relative risks and chi-squared tests at each time-point. Variables, including pregnancy and any re-intervention, will also be explored using standard time-to-event analysis methods (log-rank test). All analyses will be performed using the intention-to-treat principle, with effect sizes presented as point estimates and corresponding 95% confidence intervals. A more comprehensive analysis plan will be made available for review by the Data Monitoring Committee (DMC).

An interim report, including the analysis of major endpoints, will be provided in strict confidence to the Data Monitoring and Ethics Committee at intervals of at least 12 months, or as requested by the DMC.

#### Handling missing data

In the first instance analysis will be completed on received data, with every effort made to follow-up participants (even after protocol treatment violation) to minimise any potential bias. Sensitivity analysis of the primary outcome measure, including imputed values for missing responses, will be performed to determine the robustness of the results obtained. Methods based on multiple imputation will be used.

#### Subgroup analysis

Subgroup analyses are limited by statistical power and can produce spurious results, particularly if numerous analyses are undertaken. For this reason, any subgroup analysis will be limited to the minimisation variables listed in the Initial assessment and randomisation section. Effects within subgroups will only be investigated further if suitable tests for interaction (by including the relevant interaction parameter in the regression model) are statistically robust.

#### Economic evaluation of embolisation compared with myomectomy

An economic evaluation will be conducted alongside the clinical trial to determine the relative cost-effectiveness of UAE compared to myomectomy from the perspective of the NHS and the personal social services [13]. Health utility values associated with each arm of the trial will be calculated from the responses to the EQ-5D questionnaire. Information relating to healthcare resource use associated with the procedures and associated complications and events during the trial period will be collected. Unit costs for all healthcare resource use will be obtained from routinely collected data and the literature to estimate the total costs associated with resource use in each arm of the trial. All costs and quality-adjusted life years (QALYs) will be discounted at the currently recommended rate. Cost-utility analyses will be carried out at two and four years post-randomisation.

Regression analysis will be used to adjust the estimates for relevant covariates, in conformity with the clinical analysis. Cost-effectiveness will be expressed as incremental cost per QALY gained. The 95% confidence limits for the difference in mean costs between arms and for the incremental cost-effectiveness ratios will be calculated using non-parametric bootstrap methods. In addition, the long-term cost-effectiveness of the intervention will be estimated by modelling. A probabilistic decision model will be developed to estimate the subsequent health status and costs beyond the period of the trial until the woman reaches menopause. The model will be populated primarily by data extrapolation from this trial. Extensive sensitivity analyses will be conducted to explore areas of structural uncertainty in the analyses. Parameter uncertainty, including that relating to heterogeneity of the pre-specified subgroups, will be handled by using probabilistic sensitivity analysis and presented using cost-effectiveness curves.

#### Sample size

The sample size for the trial is based around the primary outcome measure of the total HRQL score taken from the UFS-QoL questionnaire, and is informed by the results of the pilot study at St George’s Hospital [11]. The mean difference seen here from the 118 patients who provided questionnaire responses at twelve months was 12 points in favour of myomectomy, with a standard deviation of 22 points (using an analysis of covariance approach). This is equivalent to approximately 0.5 standard deviations (rounded downwards to be conservative). To detect a difference of this size (a moderate effect [14]) with 90% power (*P* = 0.05) would require 86 patients in each group (172 in total). Whilst the number of women expected to become pregnant before the primary outcome time of two years is expected to be low (10% or less), some provision needs to be made for these women as not all questions on the UFS-QoL will be still relevant. Additionally, a further 10% has also been added to account for loss to follow-up (including withdrawals). Thus, the final target sample size has been inflated to 216 patients.

### Data management and quality assurance

#### Data protection

Data will be stored and analysed in accordance with the Data Protection Act 1998, plus any other relevant legislation. Completed forms returned to the trials office will be stored securely in a locked filing cabinet within a safe haven office. There is a strictly enforced hierarchical access policy which ensures that research staff (including those at local sites) only have access to the electronic information which is necessary for them to perform their duties, for example, allowing patients to be contacted for follow-up or allowing data checks and validation.

#### Confidentiality

Personal and sensitive data will be collected directly from trial participants and their hospital notes. Participants will be informed about the transfer of this information to the FEMME study office at Birmingham Clinical Trials Unit (BCTU) and asked for their consent. With the patient’s consent, their full name, date of birth, NHS or Community Health Index number, address, postcode, hospital number, GP details, and the participant’s telephone number and e-mail address will be collected at trial entry. This will allow direct contact with the participant and enable tracing of non-responders to assist with long-term follow-up. Patients will be identified using only their unique trial number to verify identify on the data collection forms and in any correspondence between the FEMME study office and the participating site. Consent forms will be collected by the FEMME study office and stored securely in the trials master file. These forms will be available to various regulatory bodies for inspection upon request. These data will be entered onto a secure computer database, either directly by the local site via the internet using secure socket layer (SSL) encryption technology, or indirectly from paper forms by FEMME study office staff. Access control will ensure that local trials staff will only be able to view information relating to participants at their site.

Blood samples, which have been transferred from local centres to the University of Birmingham’s Human Tissue Repository will only be identified by a code containing the participant’s trial number, the treatment they were randomized to, the time-point at which the bloods were taken, and whether the samples were drawn on days two, three, or four of the participant’s menstrual cycle. Central laboratory staff will not have access to personal or clinical trial data.

All personal information received in a paper format for the trial will be held securely in locked filing cabinets in a safe haven office and treated as strictly confidential according to BCTU policies. All staff involved in the FEMME study, be they clinical, academic, or employees of BCTU, share the same duty of care to prevent unauthorised disclosure of personal information. No data that could be used to identify an individual will be published. Personal data recorded on all documents will be regarded as strictly confidential and will be handled and stored in accordance with the Data Protection Act 1998 and any amendments.

Ethical requirements: Multicentre ethical approval for the FEMME trial was granted by the Coventry and Warwickshire REC on 15 June 2011 (reference number: 11/WM/0149). The study will be performed in accordance with the principles stated in the Declaration of Helsinki [15]. Study procedures will be guided by the standards outlined in the Guidelines for Good Clinical Practice in Clinical Trials [16].

#### Monitoring of all types of adverse event

All types of adverse event which occur to participants in the FEMME trial will be reported as soon as possible after the clinician becomes aware of the event. An adverse events form should be completed as fully as possible and sent via fax to the trials office. This form will include an assessment of causality. Upon receipt, the trials office will log details of the adverse event on that patient’s record on the central trials database and forward an anonymised copy of the form to the sponsor and the Chief Investigator. Anonymised details of all adverse events will be presented to the DMC for scrutiny each time they meet. These anonymised details will be reported to the REC annually.

### Trial administration

#### Sponsor

The University of Oxford, Wellington Square, Oxford, OX1 2JD.

#### Trial Management Group

The Trial Management Group (TMG) is responsible for the overall design and conduct of the study, analysis of the data, and reporting and dissemination of results. It will act on the advice of the Trial Steering Committee (TSC), the DMC, and the funders (National Institute for Health Research Health Technology Assessment Programme [NIHR HTA]).

#### Membership of the Trial Management Group

Professor Klim McPherson (University of Oxford) is the Chair and Chief Investigator. Professor Isaac Manyonda (St. George’s Hospital, London), Professor Mary-Ann Lumsden (Glasgow Royal Infirmary), Professor Jonathan Moss (Gartnavel Hospital, Glasgow), and Professor Anna-Maria Belli (St. George’s Hospital, London) are Clinical Lead Investigators. Dr Jane Daniels (Birmingham Clinical Trials Unit, Birmingham) and Mr Lee Middleton (Birmingham Clinical Trials Unit, Birmingham) are responsible for trial management. Professor Olivia Wu (University of Glasgow) is responsible for health economics.

#### Trial Steering Committee

The TSC will provide independent overall supervision of the trial. The members of the FEMME TSC are: Professor Siladityta Bhattacharya (Chair; Aberdeen Royal Infirmary) Professor Hilary Critchley (University of Edinburgh), Dr Tony Nicholson (Leeds Teaching Hospital N.H.S. Trust), and Ms Allison Hirst (University of Oxford).

#### Data Monitoring Committee

The primary role of the DMC is to ensure patient safety and treatment efficacy whilst the trial is ongoing. At intervals specified by the DMC the senior trial statistician will provide confidential interim analysis of all available data, alongside anonymised reports of any adverse events suffered by participants in the trial. This will be reviewed by the DMC who will advise the TSC if the trial should continue as it is, continue with modifications, or be halted due to futility, safety concerns, or overwhelming benefit. The TSC will then decide whether to close or modify any part of the trial. Unless this happens then no one other than the trial statisticians (who supplied the confidential analysis) and the members of the DMC will be made aware of the results of the interim analysis.

The members of the FEMME DMC are: Professor Doug Altman (Chair; University of Oxford), Professor Jim Thornton (Queens Medical Centre, Nottingham), and Dr. John Reidy (London).

### Trial status

The FEMME trial is currently recruiting.

## Abbreviations

AMH: Anti-Mullerian hormone
BCTU: Birmingham Clinical Trials Unit
DMC: Data Monitoring Committee
EQ-5D: EuroQol – 5D score and visual analogue scale score
GP: General practitioner
HMB: Heavy menstrual bleeding
HRQoL: Health-related quality of life
NHS: National Health Service
PBAC: Pictorial Blood loss Assessment Chart
PIS: Participant information sheet
QALY: Quality-adjusted life year
QoL: Quality of life
REC: Research Ethics Committee
SSL: Secure socket layer
TMG: Trial Management Group
UAE: Uterine artery embolization
UFS-QoL: Uterine fibroid symptom quality of life.
